# Deconditioned, disabled, or debilitated? Formalizing management of functional mobility impairments in the medical inpatient setting

**DOI:** 10.1002/jhm.12910

**Published:** 2022-07-11

**Authors:** Maylyn Martinez, Jason R. Falvey, Adam Cifu

**Affiliations:** ^1^ Department of Medicine, Section of Hospital Medicine University of Chicago Chicago Illinois USA; ^2^ Department of Physical Therapy and Rehabilitation Science University of Maryland School of Medicine Baltimore Maryland USA; ^3^ Department of Epidemiology and Public Health University of Maryland School of Medicine Baltimore Maryland USA; ^4^ Department of Medicine, Section of General Internal Medicine University of Chicago Chicago Illinois USA

## INTRODUCTION

Each year, tens of millions of patients are admitted to US hospitals.^1^ Among older adults who are hospitalized, 30%–50% will become unable to complete one or more activities of daily living (ADLs) after discharge—a condition termed hospital‐associated disability (HAD).^2^, ^3^ Over a quarter of those with HAD will experience prolonged in ADL disability.^4^ Older adults with low mobility during hospitalization are six times more likely to be institutionalized at the time of discharge and 34 times more likely to die.^5^ In spite of these detrimental, life‐altering, and sometimes permanent effects, hospitalists remain largely unaware of how to manage HAD and other addressable but often overlooked antecedent functional mobility impairments that can occur during acute hospitalization. So how can we begin to formalize our understanding and management of these common, yet nuanced clinical conditions? The answer may be easier than you think: we treat them like any other disease we manage in the hospital. For inpatient functional mobility impairments this will require clarifying (1) terminology, (2) risk assessment and diagnostics, and (3) treatment strategies.

## IS IT TIME FOR A CLINICAL GUIDELINE?

Imagine a 78‐year‐old patient with hypertension, well controlled insulin‐dependent diabetes, and history of hip replacement surgery 3 years ago is admitted to your service for community‐acquired pneumonia requiring 2 L of supplemental oxygen. The patient is independent with ADLs prior to admission and ambulates without an assistive device. After 5 days, the patient is on room air and planned for discharge. However, the nurse notes that the patient has been in bed nearly 100% of the time despite not being considered a fall risk. She is now requiring significant assistance just to get out of bed. You consult physical and occupational therapy for disposition recommendations and they suggest subacute rehabilitation due to development of HAD and physical deconditioning.

Disability leading to a nonhome discharge represents a poor outcome for a patient who was independent prior to admission and, with proper risk assessment and mobilization, may have returned home at discharge. However, since HAD and physical deconditioning are not commonly thought of as medical conditions, they can be forgotten while addressing primary problems during hospitalization. This is compounded by the absence of standardized guidance on how to recognize, diagnose, or prescribe treatment for functional mobility impairments in the medical acute care setting. Therefore, our primary purpose is to propose the development of a clinical practice guideline (CPG) for the “Management of HAD and Physical Deconditioning in Patients Hospitalized for Acute Medical Illness” to initiate a paradigm shift in how we prioritize, diagnose, and intervene upon functional mobility impairments during hospitalization.

It is critical that the CPG be evidence‐based and free from bias in the process of its creation and within its evidence base. We did not complete a formal literature review, but employed the Appraisal of Guidelines for Research and Evaluation (AGREE II) instrument^6^ to guide our assessment of whether there is data to support a medical inpatient mobility CPG. The instrument's “Rigour of Development” domain states that there be “an explicit link between the recommendations and the supporting evidence.” We, therefore, developed the following framework to outline ideal management and areas in need of evidence to support a CPG:


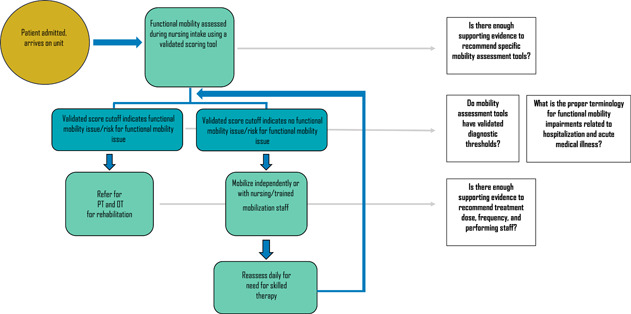


## TERMINOLOGY

### Which terms should be used to describe functional mobility impairments in the hospital?

This may be the most important step toward compelling clinicians to see these conditions as diseases that require attention during hospitalization. Functional mobility impairments are alternatively and inconsistently labeled as physical deconditioning, HAD, debility, functional decline, generalized weakness, and more. However, HAD is a condition specifically related to hospitalization and can reasonably be applied to a decrease in abilties related to acute illness and bedrest. For consistency, we will refer to acute ADL impairments as “HAD” since an impairment in one's ability to care for oneself may be considered a “disability” and development in the setting of acute illness while in the hospital implies “hospital‐associated,” making this term quite precise. New impairments in physical performance will be referred to as “physical deconditioning.” These may or not be the exact terms used by an expert panel in a CPG but making this distinction would be a crucial first step for guideline developers.

## POPULATION

### Who should be assessed?

For patients hospitalized for acute medical conditions, there are no validated scores that combine risk factors to create an overall risk score for development of HAD or physical deconditioning. There is a large body of literature describing risk factors for HAD and many can be assessed at the time of admission. Age is one major risk factor^3^ but cognitive and mood disorders can also confer very high risk.^7^, ^8^, ^9^ A patient's level of function prior to admission and their need for assistive devices with ambulation can be clues to whether they require mobility assessment as these are also potent risk factors.^10^, ^11^ Most experts agree that mobility assessment should be done for all patients at the time of admission since bedrest is harmful for nearly everyone. It is likely that guideline developers would make this recommendation based on “expert opinion.” Future research focusing on prediction scores for development of HAD or physical deconditioning could strengthen this recommendation by adding a validated screening tool. Screening, however, would likely continue to be recommended in all hospitalized patients.

## DIAGNOSIS

### How do we diagnose HAD and physical deconditioning?

In 2018, the American Geriatrics Society released a white paper^12^ stating their recommendations for mobility assessment in older adults, which highlighted 14 validated tools for evaluating functional mobility and physical performance in older patients. Of these, seven were validated in the acute care setting and, five took only a few minutes to complete and, importantly, could be performed by either rehabilitation therapists or nursing staff. Two of these assessments, the Activity Measure Post‐Acute Care (AM‐PAC)^13^ score and the Johns Hopkins Highest Level of Mobility (JH‐HLM) score, have since had further evidence supporting their reliability and construct validity for assessments done by nurses and therapists.^14^ The AM‐PAC score would thus be important to include in a CPG for HAD diagnosis while physical performance tools such as JH‐HLM and even gait speed,^15^ which is validated and has established diagnostic cutpoints, may be used for diagnosis of physical deconditioning. Future research may then focus on the predictive precision of the cutpoints that are used as thresholds to prescribe varying intensities of treatment.

## TREATMENT

### How do we treat functional mobility impairments in the medical acute care setting?

The best way to treat and prevent HAD and physical deconditioning is with mobilization beginning early in admission. This can be accomplished by physical and occupational therapists, nursing staff, or other properly trained staff. There are myriad studies describing the association of early mobilization and rehabilitation of ADL loss with decreases in length‐of‐stay, functional decline, institutionalization, disability, and mortality in hospitalized patients.^16^, ^17^, ^18^, ^19^, ^20^, ^21^ As with other medical conditions, treatment should be tailored to the patient and consider “severity of illness.” There is also longstanding evidence that amount and duration of rehabilitation matter,^22^, ^23^ so an optimal CPG would not only recommend mobilization but would specify “dose,” “frequency,” and “mode of delivery” (e.g., skilled therapy vs. ambulate with nursing staff).

## IF NOT NOW, THEN WHEN?

One might argue that we don't yet have enough evidence to support official recommendations. But given the existing literature on diagnosis, treatment, and consequences of functional mobility impairments, it seems unacceptable that hospitalists are given no systematic way to manage these conditions. Imagine if all elderly patients admitted for sepsis due to a urinary tract infection received a cardiology consult to ensure they were not inadvertently discharged to home with any cardiac conditions. Because of the lack of understanding of functional mobility impairments, physical or occupational therapists are frequently consulted in this way. Their dual responsibility as therapists administering treatment and experts providing recommendations for safe discharge levels of care means therapists are frequently saddled with triaging, diagnosing, and treating mobility issues regardless of a patient's functional status. In fact, PT referrals occur for hospitalized patients with no need for skilled therapy as much as 38% of the time.^24^ But given they are a constrained resource in most hospital settings, like other consultants, their services should be reserved for the appropriate patients. Targeting allocation of skilled therapy to vulnerable patients is key for treating and preventing HAD and physical deconditioning in hospitalized patients but cannot be achieved with the current standards of practice.

It is possible that current evidence supports only level 2B recommendations (weak recommendation, moderate‐quality evidence). However, any guidelines could significantly improve how hospitalists manage functional mobility impairments. They would also serve to focus future research, getting us closer to 1A recommendations (strong recommendation, high‐quality evidence). It is simply critical that we comprehend that our patients present with and develop distinct “mobility maladies” and “ability ailments” related to their acute illness and hospitalization. Formal framing of these conditions and their life‐changing effects will help clinicians begin to manage them like other conditions, which is an essential step toward home and functional independence for our patients.

## CONFLICT OF INTEREST

The authors declare no conflict of interest.
